# Genetic loci associated with prevalent and incident myocardial infarction and coronary heart disease in the Cohorts for Heart and Aging Research in Genomic Epidemiology (CHARGE) Consortium

**DOI:** 10.1371/journal.pone.0230035

**Published:** 2020-11-13

**Authors:** Julie Hahn, Yi-Ping Fu, Michael R. Brown, Joshua C. Bis, Paul S. de Vries, Mary F. Feitosa, Lisa R. Yanek, Stefan Weiss, Franco Giulianini, Albert Vernon Smith, Xiuqing Guo, Traci M. Bartz, Diane M. Becker, Lewis C. Becker, Eric Boerwinkle, Jennifer A. Brody, Yii-Der Ida Chen, Oscar H. Franco, Megan Grove, Tamara B. Harris, Albert Hofman, Shih-Jen Hwang, Brian G. Kral, Lenore J. Launer, Marcello R. P. Markus, Kenneth M. Rice, Stephen S. Rich, Paul M. Ridker, Fernando Rivadeneira, Jerome I. Rotter, Nona Sotoodehnia, Kent D. Taylor, André G. Uitterlinden, Uwe Völker, Henry Völzke, Jie Yao, Daniel I. Chasman, Marcus Dörr, Vilmundur Gudnason, Rasika A. Mathias, Wendy Post, Bruce M. Psaty, Abbas Dehghan, Christopher J. O’Donnell, Alanna C. Morrison

**Affiliations:** 1 Human Genetics Center, Department of Epidemiology, Human Genetics, and Environmental Sciences, School of Public Health, The University of Texas Health Science Center at Houston, Houston, Texas, United States of America; 2 Office of Biostatistics Research, National Heart, Lung, and Blood Institute, National Institutes of Health, Bethesda, Maryland, United States of America; 3 Framingham Heart Study, National Heart, Lung, and Blood Institute, National Institutes of Health, Framingham, Massachusetts, United States of America; 4 Cardiovascular Health Research Unit, Department of Medicine, University of Washington, Seattle, Washington, United States of America; 5 Department of Epidemiology, Erasmus University Medical Center, Rotterdam, The Netherlands; 6 Division of Statistical Genomics, Department of Genetics, Washington University School of Medicine, St. Louis, Missouri, United States of America; 7 GeneSTAR Research Program, Department of Medicine, Johns Hopkins University School of Medicine, Baltimore, Maryland, United States of America; 8 Interfaculty Institute for Genetics and Functional Genomics, The University Medicine and Ernst-Moritz-Arndt-University Greifswald, Greifswald, Germany; 9 DZHK (German Centre for Cardiovascular Research), partner site Greifswald, Greifswald, Germany; 10 Division of Preventive Medicine, Brigham and Women’s Hospital, Boston, Massachusetts, United States of America; 11 Icelandic Heart Association, Kovapvogur, Iceland; 12 Faculty of Medicine, University of Iceland, Reykajvik, Iceland; 13 The Institute for Translational Genomics and Population Sciences, Department of Pediatrics, The Lundquist Institute for Biomedical Innovation at Harbor-UCLA Medical Center, Torrance, California, United States of America; 14 Department of Biostatistics, The University of Washington, Seattle, Washington, United States of America; 15 Human Genome Sequencing Center, Baylor College of Medicine, Houston, Texas, United States of America; 16 Department of Internal Medicine, Erasmus University Medical Center, Rotterdam, The Netherlands; 17 Laboratory of Epidemiology and Population Sciences, Intramural Research Program, National Institute on Aging, National Institutes of Health, Bethesda, Maryland, United States of America; 18 Department of Epidemiology and Biostatistics, Imperial College London, London, United Kingdom; 19 Department of Internal Medicine B - Cardiology, Pneumology, Infectious Diseases, Intensive Care Medicine, The University Medicine Greifswald, Greifswald, Germany; 20 Department of Medicine and Epidemiology, Johns Hopkins University School of Medicine, Baltimore, Maryland, United States of America; 21 Harvard Medical School, Boston, Massachusetts, United States of America; 22 Division of Cardiology, Department of Medicine, University of Washington, Seattle, Washington, United States of America; 23 Department of Epidemiology, Harvard T.H. Chan School of Public Health, Boston, Massachusetts, United States of America; 24 Institute for Community Medicine, University Medicine Greifswald, Greifswald, Germany; 25 Department of Epidemiology, The University of Washington, Seattle, Washington, United States of America; 26 Department of Health Services, The University of Washington, Seattle, Washington, United States of America; 27 Kaiser Permanente Research Institute, Seattle, Washington, United States of America; 28 VA Boston Healthcare System, Veteran’s Affair, Boston, Massachusetts, United States of America; 29 Cardiovascular Medicine Division, Brigham and Women’s Hospital, Boston, Massachusetts, United States of America; Universite de Montreal, CANADA

## Abstract

**Background:**

Genome-wide association studies have identified multiple genomic loci associated with coronary artery disease, but most are common variants in non-coding regions that provide limited information on causal genes and etiology of the disease. To overcome the limited scope that common variants provide, we focused our investigation on low-frequency and rare sequence variations primarily residing in coding regions of the genome.

**Methods and results:**

Using samples of individuals of European ancestry from ten cohorts within the Cohorts for Heart and Aging Research in Genomic Epidemiology (CHARGE) consortium, both cross-sectional and prospective analyses were conducted to examine associations between genetic variants and myocardial infarction (MI), coronary heart disease (CHD), and all-cause mortality following these events. For prevalent events, a total of 27,349 participants of European ancestry, including 1831 prevalent MI cases and 2518 prevalent CHD cases were used. For incident cases, a total of 55,736 participants of European ancestry were included (3,031 incident MI cases and 5,425 incident CHD cases). There were 1,860 all-cause deaths among the 3,751 MI and CHD cases from six cohorts that contributed to the analysis of all-cause mortality. Single variant and gene-based analyses were performed separately in each cohort and then meta-analyzed for each outcome. A low-frequency intronic variant (rs988583) in *PLCL1* was significantly associated with prevalent MI (OR = 1.80, 95% confidence interval: 1.43, 2.27; *P* = 7.12 × 10^−7^). We conducted gene-based burden tests for genes with a cumulative minor allele count (cMAC) ≥ 5 and variants with minor allele frequency (MAF) < 5%. *TMPRSS5* and *LDLRAD1* were significantly associated with prevalent MI and CHD, respectively, and *RC3H2* and *ANGPTL4* were significantly associated with incident MI and CHD, respectively. No loci were significantly associated with all-cause mortality following a MI or CHD event.

**Conclusion:**

This study identified one known locus (*ANGPTL4*) and four new loci (*PLCL1*, *RC3H2*, *TMPRSS5*, and *LDLRAD1*) associated with cardiovascular disease risk that warrant further investigation.

## Introduction

Coronary heart disease (CHD) is a leading cause of morbidity and mortality worldwide, accounting for one of every seven deaths in the United States in 2016 [1]. In addition to major modifiable risk factors such as dyslipidemia, hypertension, diabetes, and cigarette smoking [2], genetic susceptibility to CHD has also been investigated extensively through family-based studies, candidate gene studies, and more recently genome-wide association studies (GWAS) [3–9]. With progressively expanded sample sizes in recent GWAS, at least 160 loci have been associated with the risk of coronary artery disease [10–13]. Most of these loci are represented by common variants located in noncoding regions, resulting in limited implications for causal genes and etiological pathways. Further, while most available data are derived from genome-wide analysis of prevalent CHD, data are sparse from prospective studies of incident cardiovascular events in populations free of baseline cardiovascular disease.

Low-frequency and rare coding sequence variations across the genome have been investigated in studies of cardiovascular disease risk factors [14–18], with the goal of better understanding the etiology of these risk factors and to advance the discovery of the treatment and prevention of diseases [19]. We previously published the results from a prospective analysis of CHD among individuals of European ancestry from the Cohorts for Heart and Aging Research in Genomic Epidemiology (CHARGE) Consortium, and identified low-frequency and common variants associated with incident CHD [20].

In this current study of individuals of European ancestry, we implemented both a cross-sectional and prospective study design in the setting of the CHARGE Consortium to examine the association between low-frequency and rare genetic variants and the risk of prevalent and incident myocardial infarction (MI) and CHD. Study of incident cardiovascular events is enabled by the rigorous prospective design of population cohorts contributing to the CHARGE Consortium. We also investigated whether these genetic variants are associated with all-cause mortality after incident MI and CHD.

## Materials and methods

### Study design and participants

Ten cohorts within the CHARGE Consortium Subclinical Working Group were included in this study: Age, Gene, Environment, Susceptibility Study (AGES), Atherosclerosis Risk in Communities (ARIC) Study, Cardiovascular Health Study (CHS), Family Heart Study (FamHS), Framingham Heart Study (FHS), the GeneSTAR Study (GeneSTAR), Multi-Ethnic Study of Atherosclerosis (MESA), Rotterdam Study (RS), Study of Health in Pomerania (SHIP), and the Women’s Genome Health Study (WGHS). Detailed characteristics of the participating cohorts and study participant are shown in the S1 Document. All study participants provided written informed consent to participate in genetic studies, and all study sites received approval to conduct this research from their local Institutional Review Boards (IRB) respectively. AGES was approved by the National Bioethics Committee in Iceland that acts as the institutional review board for the Icelandic Heart Association and by the National Institute on Aging Intramural Institutional Review Board. ARIC was approved by the University of Mississippi Medical Center IRB, Wake Forest University Health Sciences IRB, University of Minnesota IRB, and John Hopkins University IRB. CHS was approved by the Wake Forest University Health Sciences IRB, University of California, Davis IRB, John Hopkins University IRB, and University of Pittsburgh IRB, and University of Washington IRB. FamHS was approved by the Washington University School of Medicine IRB. FHS was approved by the Boston University IRB. GeneSTAR was approved by the Johns Hopkins Medicine IRB. MESA was approved by the institutional review boards of the six field centers have approved the study protocol (Wake Forest University School of Medicine, University of Minnesota, Northwestern University, Columbia University, Johns Hopkins University, University of California, Los Angeles). RS was approved by the Medical Ethics Committee of the Erasmus MC and the Dutch Ministry of Health, Welfare and Sport. SHIP was approved by the Medical Ethics Committee of the University of Greifswald. WGHS was approved by the Brigham and Women’s Hospital IRB.

### Genotype calling and quality control

Participants from WGHS were genotyped by the HumanHap300 Duo+ (Illumina, Inc., San Diego, CA), and all other study participants were genotyped by the HumanExome BeadChip (v1.0–1.2, Illumina, Inc., San Diego, CA) which contains more than 240,000 variants including those discovered through exome sequencing in ~12,000 individuals and other non-coding common variants such as previously-reported GWAS signals and ancestry-informative markers. Data for AGES, ARIC, CHS, FamHS, FHS, MESA, and RS were jointly called at the University of Texas Health Science Center at Houston [21]; SHIP was called in Illumina GenomeStudio using the CHARGE Consortium joint calling cluster file; GeneSTAR used the Illumina GenomeStudio and zCall software [22]; and WGHS data was called using the Illumina BeadStudio v.3.3. Variant quality control (QC) was performed centrally [21] and by the individual studies, including checking concordance with previous GWAS data, and excluding participants with missing >5% genotypes, population clustering outliers, individuals with high inbreeding coefficients or heterozygote rates, gender mismatches, duplicated pairs, and unexpectedly high proportion of identity-by-descent sharing for family studies. Joint calling of the measured exome chip genotypes allowed for the ability to accurately genotype rare variation using array technology.

### Cardiovascular outcome definition

Two cardiovascular outcomes were examined for association in this study: 1) MI: fatal or non-fatal MI; and 2) CHD: fatal or non-fatal MI, fatal CHD, sudden death within one hour of onset of symptoms, or revascularization (percutaneous coronary artery intervention such as stent or balloon angioplasty, or coronary artery bypass grafting). No exclusions were applied for the cross-sectional analysis of prevalent MI and prevalent CHD. For analysis of incident events, participants with a history of MI, CHD or revascularization at the baseline examination were excluded. Follow-up time was defined as the time from the baseline exam to MI or CHD diagnosis, the time of death, last date of contact, or at the end of follow-up, whichever came first. All-cause mortality after MI or CHD was also investigated with follow-up time from first MI or CHD incident events until death, loss to follow-up, or the end of study.

### Statistical analysis

Single variant and gene-based analyses were conducted in each participating cohort respectively, followed by meta-analysis performed for each cardiovascular outcome to summarize results. All autosomal variants were coded to the minor allele observed in the CHARGE jointly called data [21] and assumed log-additive genetic effect in the analyses. The minor allele frequency (MAF) thresholds were defined using the European allele frequencies derived from the CHARGE jointly called data [21]. Variant annotation was performed centrally within CHARGE using dbNSFP [23, 24]. Low-frequency variants (MAF ≥ 1% and less than 5%) were included in single variant tests for prevalent MI and CHD and for incident MI. Single variant results for incident CHD followed the same analytic approach and are reported in Morrison et al. [20] and are not reported in detail here. Gene-based tests were evaluated for MI and CHD outcomes: the Sequence Kernel Association Test (SKAT) [25] was used for incident events and post-MI mortality and a burden test [26] was performed for prevalent and incident events and for post-MI mortality. Only functional coding variants (missense, stop-gain, stop-loss, or splice-site changes) with MAF < 5% were aggregated by gene, and we only analyzed genes with a cumulative minor allele count (cMAC) ≥ 5.

For both single variant and gene-based burden tests of prevalent events, we performed Firth’s logistic regression model to test the association between each variant and cardiovascular outcome using the “logistf” package in R [27–29] to account for the possible inflated type one error in the rare variant association analysis in a case-cohort study design [30]. Meta-analysis for prevalent events was conducted with METAL [31] and applied the genomic control correction. For the single variant and two gene-based tests of incident events, a Cox proportional hazards regression model implemented in the seqMeta package in R was used to test the association between each variant and the incident event or post-event all-cause mortality. SeqMeta was used both at the study-specific analysis and meta-analysis levels [32]. All study-specific analyses (single variant and gene-based tests) were adjusted for cohort-specific design variables (e.g. study sites, family structure) and for population substructure using principal components as needed. We applied a Bonferroni corrected threshold to determine statistical significance in each analysis as described below.

## Results

### Prevalent MI and CHD association

A total of 27,349 participants of European ancestry from seven cohorts including 1831 prevalent MI cases (6.7%) and 2518 prevalent CHD cases (9.2%) were used in the meta-analyses of prevalent events (S1 Table). We individually examined a total of 6,221 low-frequency variants, across all autosomal chromosomes corresponding to a Bonferroni corrected significance threshold of *P* = 8.04 × 10^−6^. A low-frequency (MAF = 1.64%) intronic variant (rs988583) in the phospholipase C like 1 gene (*PLCL1*) was significantly associated with prevalent MI (*P* = 7.12 × 10^−7^; OR = 1.80, 95% confidence interval = 1.43 to 2.27; Table 1). No low-frequency variants were significantly associated with prevalent CHD. Cohort specific summary statistics for this association is shown in S2 Table.

**Table 1:** Low-frequency variants associated with prevalent MI and CHD.

Outcome	Variant	Chromosome and Position*	Allele 1 / Allele 2**	Locus	Function	Frequency of Allele 2 (%)	Odds Ratio (95% Confidence Interval)	p-value
MI	rs988583	2:198987935	C/A	*PLCL1*	Intronic	1.64	1.80 (1.43, 2.27)	7.12×10^−7^

*Chromosome and nucleotide positions are based on genome build GRCh37.

**Effect allele.

In the gene-based burden tests, we analyzed 16,628 autosomal genes that contained functional low-frequency or rare variants with MAF < 5% and with a cumulative minor allele count (cMAC) ≥ 5; therefore, the Bonferroni corrected p-value threshold was *P* = 3.01 × 10^−6^. The transmembrane serine protease 5 gene (*TMPRSS5*) on chromosome 11, containing nine nonsynonymous rare variants (S3 Table), was significantly associated with prevalent MI (*P* = 2.59 × 10^−6^, OR = 3.00, 95% confidence interval: 1.90, 4.73; Table 2). The low-density lipoprotein receptor class A domain containing 1 gene (*LDLRAD1*) on chromosome 1 contained seven rare variants (S3 Table) and was significantly associated with prevalent CHD (*P* = 1.30 × 10^−6^, OR = 4.48, 95% confidence interval: 2.44, 8.23; Table 2).

**Table 2:** Genes associated with prevalent MI and CHD in gene-based analysis.

Outcome	Gene	Chromosome and Position*	cMAC**	Variants (n)^	Test	Odds Ratio (95% Confidence Interval)	p-value
MI	*TMPRSS5*	11:113558268–113577151	152.02	9	Burden	3.00 (1.90, 4.73)	2.59×10^−6^
CHD	*LDLRAD1*	1:54472971–54483859	60.05	7	Burden	4.48 (2.44, 8.23)	1.30×10^−6^

*Chromosome and nucleotide positions are based on genome build GRCh37.

**cMAC = overall cumulative minor allele count.

^^^Variants (n) = number of variants included in the analysis; variants were restricted to those with MAF < 5% and annotated as nonsynonymous, splice-site, or stop loss/gain function.

### Incident MI and CHD association

Nine cohorts contributed a total of 55,736 participants of European ancestry to the analyses of incident events, where 3,031 incident MI cases (5.4%) were reported during an average of 15.0 years of follow-up and 5,425 incident CHD cases (9.73%) were reported during an average of 15.6 years of follow-up (S4 Table). A total of 9,852 low-frequency autosomal variants were individually tested for association with incident MI, with adjustment of age, sex, and population substructure. The Bonferroni corrected p-value threshold for single variant analysis of incident MI was *P* = 5.08 × 10^−6^. No low-frequency variants were significantly associated with incident MI. As previously stated, single variant results for incident CHD are reported in Morrison et al. and are not reported here, but include a significant association between a low-frequency variant in *ANGPTL4* and a decreased risk of incident CHD [20].

For the gene-based analyses, we examined 17,574 genes across all autosomal chromosomes for association with incident MI, and the Bonferroni corrected significance level was *P* = 2.85 × 10^−6^. The ring finger and CCCH-Type domains 2 gene (*RC3H2*) on chromosome 9 was significantly associated with incident MI in the burden test (*P* = 2.99 × 10^−6^, OR = 0.35, 95% confidence interval = 0.23, 0.55; Table 3) and contained 12 nonsynonymous and one splice-site rare variants (S5 Table). No genes were significantly associated with incident MI using SKAT. For the gene-based analyses of incident CHD, 16,620 genes were evaluated and the Bonferroni significance levels was P = 3.01 × 10^−6^. Angiopoietin-like 4 (*ANGPTL4*) on chromosome 19 was significantly associated with incident CHD using SKAT (P = 1.29 × 10^−6^; Table 3) and contained 10 variants (S5 Table), and no gene was significantly associated using the burden test.

**Table 3:** Genes associated with incident MI and CHD in gene-based analysis.

Outcome	Gene	Chromosome and Position*	cMAC**	Variants (n)^	Test	Hazards Ratio (95% Confidence Interval)	p-value
MI	*RC3H2*	9:125606835–125667562	356.02	13	Burden	0.35 (0.23, 0.55)	2.99×10^−6^
CHD	*ANGPTL4*	19:8429011–843925	2830.07	10	SKAT	-	1.29×10^−6^

*Chromosome and nucleotide positions are based on genome build GRCh37.

**cMAC = overall cumulative minor allele count.

^^^Variants (n) = number of variants included in the analysis; variants were restricted to those with MAF < 5% and annotated as nonsynonymous, splice-site, or stop loss/gain function.

### Post MI and CHD mortality analysis

Among the 3,751 MI and CHD cases from six cohorts that contributed to the analysis of all-cause mortality, there were 1,860 all-cause deaths over a mean 10.9 years of follow-up (S6 Table). We examined 9,943 low-frequency autosomal variants in the single variant analysis (Bonferroni corrected significant level of *P* = 5.03 × 10^−6^) and 17,574 genes in the gene-based analysis (Bonferroni corrected significant level of *P* = 2.85 × 10^−6^). No single variant or gene reached the significance threshold in the analysis of all-cause mortality among survivors of MI or CHD. We examined the significant variants and genes reported in Tables 1–3 for their relationship with mortality following a MI or CHD event, and none of them were significantly associated with all-cause mortality (S7 Table).

## Discussion

Our study evaluated genetic susceptibility to MI and CHD in cross-sectional and prospective settings among individuals of European ancestry. We identified one new locus associated with prevalent MI, and also investigated disease risk in the context of gene-based analyses.

Single variant analysis of prevalent cardiovascular outcomes revealed a low-frequency (MAF = 1.64%) intronic variant, rs988583, in *PLCL1* significantly associated with increased risk of MI (*P* = 7.12 × 10^−7^). *In silico* replication was conducted by a look up of rs988583 and its association with prevalent MI in the Myocardial Infarction Genetics and CARDIoGRAM exome chip meta-analysis public release [33], and there was no significant association with MI (*P* = 0.34). A GWAS of MI and coronary artery disease (CAD) in a Saudi Arab population identified an intergenic variant, rs7421388, near *PLCL1* associated with CAD (*P* = 4.31 × 10^−6^) and replicated in an independent sample of Saudi Arabs (*P* = 5.37 × 10^−7^) [34]. In another study of an ethnic Arab population, rs1147169 in *PLCL1* was protective against a low level of high density lipoprotein-cholesterol levels (*P* = 2.87 × 10^−7^) [35]. In individuals of European ancestry, rs988583 and rs1147169 are in linkage equilibrium (R^2^ = 0.0043). In addition to these studies, *PLCL1* has been implicated in coronary artery aneurysm in Kawasaki disease and *PLCL1* might play a role in the regulation of vascular endothelial cell inflammation via interference with proinflammatory cytokine expression [36].

A burden test aggregating low-frequency and rare coding variants in genes showed a significant positive association between *TMPRSS5* and prevalent MI (*P* = 2.59 × 10^−6^) and *LDLRAD1* and prevalent CHD (P = 1.30 × 10^−6^), and a significant protective association between *RC3H2* and incident MI (*P* = 2.99 × 10^−6^). A significant association between *ANGPTL4* and incident CHD was identified using SKAT (P = 1.29 × 10^−6^). The relationship between *ANGPTL4* and CHD has been previously reported, with the rs116843064 missense variant playing a major role in reducing lipid levels and risk of CHD [33, 37]. Serine proteases, such as *TMPRSS5*, are known to be involved in many physiological and pathological processes, and *TMPRSS5* has been implicated in impaired hearing function [38]. Little is known about *LDLRAD1*, with most marked gene expression in lung and fallopian tube [39], and a rare variant in this gene has been associated with breast cancer [40]. Roquin-2 is encoded by *RC3H2* and has been shown to play a key role in posttranscriptional regulation of autoimmunity and inflammatory response [41]. Each of these genes associated with prevalent or incident cardiovascular outcomes has rare and low-frequency variants underlying the gene burden tests (S3 and S5 Tables). We identified 11 putative driving variants of these gene-based associations (i.e. those with p<0.05 in S3 and S5 Tables; rs201233178, rs200417674, and rs116913282 in *TMPRSS5*; rs150560713, rs202234131, rs142900519, and rs76122098 in *LDLRAD1*; rs201920127, rs144714368, and rs199901510 in *RC3H2*; and rs116843064 in *ANGPTL4*). An *in silico* replication was not possible due to the rare frequency of these coding variants and their absence in the public release of the Myocardial Infarction Genetics and CARDIoGRAM exome chip meta-analysis or the analysis of CAD in the UK Biobank and the UK Biobank and CARDIoGRAMplusC4D meta-analysis [10, 33]. However, it is important to note that rs116843064 of *ANGPTL4* is the same variant found in the single variant analysis conducted for incident CHD by Morrison et al., and this gene is likely to be driving the significant association found in the SKAT analysis of incident CHD [20]. It is of interest that the effect size of the gene-based tests (Tables 2 and 3) are larger than the single variant test effect size (Table 1). This shows that aggregate tests of rare variants indeed have a larger effect on disease outcomes, although there remains some scientific debate regarding the utility of conducting aggregate tests of rare variants.

Although there was no statistically significant result found for all-cause mortality after MI or CHD, after accounting for multiple testing, the protective direction of effect for the mortality results suggests that genetic variants might contribute differently in various stages of disease manifestation. Given the limited statistical power of our findings for post-event survival, our study supports the need for substantially larger well-phenotyped cohorts to differentiate effects of variants associated with CHD from post-event mortality. Another limitation that may reside within mortality analysis is that there may have been a presence of index event bias, which arises from selecting a population on the basis of a prior, or an “index” event [42]. It is possible that due to this selection bias, individuals with MI or CHD presented modestly lower rate of mortality compared to those without disease endpoints. This is a major challenge affecting prognosis research. Several approaches are under development that aim to mitigate this type of bias, such as the “Slope-Hunter” method proposed by Mahmoud et al [43]. This method utilizes clustering technique to ultimately identify variants that only affect prognosis, and also to find estimated adjustment factors by identifying variants that affect incidence. With more implementation in the future, this may be suitable to be applied in future investigation. Also, generally the loci identified for prevalent disease were not the same as those identified for incident disease (S7 Table), as has been observed in previous studies [9]. A possible explanation for these observed differences is that genetic studies of cardiovascular diseases are usually conducted with the cross-sectional study design, which has the potential to oversample participants with longer post-event survival [44] and the results do not always replicate in the prospective studies for disease onset and vice versa [9].

An advantage of this study is that within the setting of the CHARGE Consortium we are able to evaluate and make comparisons between cross-sectional and prospective study designs, and to investigate all-cause mortality following cardiovascular events. There are differing, but overlapping, sample sizes across the various study designs: 27,349 participants from seven cohorts for prevalent outcomes, 55,736 participants from nine cohorts for incident outcomes, and 3,751 MI and CHD cases from six cohorts that contributed to the analysis of all-cause mortality. These differing sample sizes influence our power to detect associations, and inferences about similarities and differences across study designs could be due to biological differences or differences in sample sizes. This investigation of low-frequency and rare variants was limited to the variants included on the genotyping platforms (HumanHap300 Duo+ and HumanExome BeadChip, v1.0–1.2, Illumina, Inc., San Diego, CA) and was also limited to individuals of European ancestry. Additionally, although the variants on the genotyping platform and included in our gene-based tests were enriched for coding variants predicted to be causal, we cannot attribute causality to the variants or genes with novel associations. A strength of this study is that the quality of rare variant genotype calling was maximized by the joint clustering performed within CHARGE on thousands of samples [21].

In conclusion, this study comprehensively evaluated the relationship between autosomal genetic variation and prevalent and incident cardiovascular outcomes in participants of European ancestry in the context of the CHARGE consortium. We identified one known locus (*ANGPTL4*) and four new loci (*PLCL1*, *RC3H2*, *TMPRSS5*, and *LDLRAD1*) associated with cardiovascular disease risk that warrant further investigation.

## Supporting information

S1 Document Characteristics of the participating cohorts. (DOCX)

S1 Table Study participants’ characteristics for prevalent MI and CHD analysis. (XLSX)

S2 Table Cohort specific summary statistics for low-frequency variant associated with prevalent MI. (XLSX)

S3 Table Low-frequency and rare variants underlying top signals from gene-based analysis for prevalent events. (XLSX)

S4 Table Study participants’ characteristics for incident MI and CHD analysis. (XLSX)

S5 Table Low-frequency and rare variants underlying top signals from gene-based analysis for incident events. (XLSX)

S6 Table Study participants’ characteristics for post MI and CHD mortality analysis. (XLSX)

S7 Table Prevalent and incident findings in relation to corresponding outcomes. (XLSX)
