# Supplementary material for: Genetic loci associated with prevalent and incident myocardial infarction and coronary heart disease in the Cohorts for Heart and Aging Research in Genomic Epidemiology (CHARGE) Consortium
Source: PLoS One. 2020 Nov 13;15(11):e0230035. doi: 10.1371/journal.pone.0230035 (PMC7665790; doi:10.1371/journal.pone.0230035)
Supplement: S1 Document — (DOCX) [file pone.0230035.s001.docx]

**S1 Document: Characteristics of the participating cohorts.**

**The Age, Gene, Environment, Susceptibility Study (AGES)**

The Reykjavik Study cohort originally comprised a random sample of 30,795 men and women born in 1907–1935 and living in Reykjavik in 1967. 2 A total of 19381 attended, resulting in 71% recruitment rate. The study sample was divided into six groups by birth year and birth date within month. One group was designated for longitudinal follow-up and was examined in all stages. One group was designated a control group and was not included in examinations until 1991. Other groups were invited to participate in specific stages of the study. Between 2002 and 2006, the AGES-Reykjavik study re-examined 5764 survivors of the original cohort who had participated before in the Reykjavik Study.

**Atherosclerosis Risk in Communities (ARIC) study**: The ARIC study is a population-based prospective cohort study of cardiovascular disease sponsored by the National Heart, Lung, and Blood Institute (NHLBI). ARIC included 15,792 individuals, predominantly European American and African American, aged 45-64 years at baseline (1987-89), chosen by probability sampling from four US communities (Forsyth County, NC; Jackson, MS; suburbs of Minneapolis, MN; and Washington County, MD). Cohort members completed three additional triennial follow-up examinations, a fifth exam in 2011-2013, a sixth exam in 2016-2017, and a seventh exam in 2018-2019. The ARIC study has been described in detail previously [PMID: 2646917]. Prevalent MI and CHD events were determined by self-report at the baseline examination. MI incidence was ascertained by reviewing death certificate and hospital discharge records, contacting participants annually, and identifying hospitalizations and deaths during the prior year [PMID: 8606324]. Incident MI events occurred on or before December 31, 2011.

**The Cardiovascular Health Study (CHS)**

The Cardiovascular Health Study (CHS) is a population-based cohort study of risk factors for coronary heart disease and stroke in adults ≥65 years conducted across four field centers [PMID: 1669507]. The original predominantly European ancestry cohort of 5,201 persons was recruited in 1989-1990 from random samples of the Medicare eligibility lists; subsequently, an additional predominantly African-American cohort of 687 persons were enrolled for a total sample of 5,888.

Blood samples were drawn from all participants at their baseline examination and DNA was subsequently extracted from available samples. Genotyping was performed at the General Clinical Research Center’s Phenotyping/Genotyping Laboratory at Cedars-Sinai among CHS participants who consented to genetic testing and had DNA available.

CHS was approved by institutional review committees at each field center and individuals in the present analysis had available DNA and gave informed consent including consent to use of genetic information for the study of cardiovascular disease.

**The Family Heart Study (FamHS)**

The Family Heart Study (FamHS) is a multicenter, population-based, family study designed to investigate the determinants of cardiovascular disease. The collection of phenotypes and covariates as well as clinical examination have been previously described [https://dsgweb.wustl.edu/fhscc/, PMID: 8651220]. In brief, the FamHS began in 1992 with the ascertainment of 1,200 families, half randomly sampled and half selected because of an excess of coronary heart disease (CHD) or risk factor abnormalities as compared with age- and sex-specific population rates. The families, with approximately 6,000 European descent subjects, were sampled from four population-based parent studies: the Framingham Heart Study, the Utah Family Tree Study, and two centers for the ARIC study. The participants attended a clinic visit between the years 1994-1996 and a broad range of phenotypes was assessed in the general domains of CHD, atherosclerosis, cardiac and vascular function, inflammation and hemostasis, lipids and lipoproteins, blood pressure, diabetes and insulin resistance, pulmonary function, diet, habitual physical activity, anthropometry, medication use, cancer and medical history. Approximately 8 years later, 2,756 subjects belonging to the 510 of the largest and most informative pedigrees were invited for a second clinical exam (2002-2004). The most important CHD risk factors were measured again.

**The Framingham Heart Study (FHS)**

Framingham Heart Study (FHS) is a community-based prospective study designed to investigate the incidence of cardiovascular diseases and factors related to its development. Study population was composed of three cohorts with European ancestry, original (5,209 participants ascertained systematically from two-thirds of the households in the town of Framingham, MA, beginning in 1948), Offspring (5,124 children of the original cohort, and spouses of those children, beginning in 1972), and the Third Generation (4,095 children of the Offspring cohort, beginning in 2002). In addition to the initial examination and face-to-face interview, participants were invited to follow-up examinations at the study clinics that were accomplished every two years for the original cohort or every four years for the offspring and gen3 cohorts. An un-related spouse cohort was formed in 2004 include 103 spouses of the offspring participants. [PMID: 13751193, 1208363]

**The GeneSTAR Study (GeneSTAR)**

GeneSTAR is a family-based prospective study of risk factors, occult disease, and incident cardiovascular disease in siblings, later extended to offspring and whole pedigrees. European- and African American families were identified from probands with early-onset (< age 60) coronary artery disease (CAD) hospitalized in any of 10 Baltimore hospitals between 1983 and 2007. Siblings completed baseline screening between 1983 and 2007, while offspring, co-parents of the offspring, and additional siblings completed baseline screening between 2003 and 2007. Siblings and offspring are followed every 5 years for cardiovascular and other comorbid incident events. DNA was collected at baseline (1991-2007) or follow-up (for those whose baseline visit was pre-1991). Probands were not eligible if they had CAD associated with calcific aortic stenosis or chronic glucocorticosteroid therapy, following organ transplantation or post intensive chest radiation, had an autoimmune disease like systemic lupus, or if they had a cocaine-induced myocardial infarction. Participants younger than age 21 or older than age 80, who had known CAD or an autoimmune disease such as systemic lupus, were taking chronic glucocorticosteroids, had undergone any organ transplantation, or had major comorbidity that had a life expectancy under 5 years of age were excluded. The full sample includes 4423 participants, 51% female/49% male, and 38% African American/61% European American/1% other American. [PMID: 17950799, 25066685]

**The Multi-Ethnic Study of Atherosclerosis (MESA)**

The Multi-Ethnic Study of Atherosclerosis (MESA) is a study of the characteristics of subclinical cardiovascular disease and the risk factors that predict progression to clinically overt cardiovascular disease or progression of the subclinical disease. MESA consisted of a diverse, population-based sample of an initial 6,814 asymptomatic men and women aged 45-84. 38 percent of the recruited participants were white, 28 percent African American, 22 percent Hispanic, and 12 percent Asian, predominantly of Chinese descent. Participants were recruited from six field centers across the United States: Wake Forest University, Columbia University, Johns Hopkins University, University of Minnesota, Northwestern University and University of California - Los Angeles. Each participant received an extensive physical exam and determination of coronary calcification, ventricular mass and function, flow-mediated endothelial vasodilation, carotid intimal-medial wall thickness and presence of echogenic lucencies in the carotid artery, lower extremity vascular insufficiency, arterial wave forms, electrocardiographic (ECG) measures, standard coronary risk factors, sociodemographic factors, lifestyle factors, and psychosocial factors. Selected repetition of subclinical disease measures and risk factors at follow-up visits allowed study of the progression of disease. Participants are being followed for identification and characterization of cardiovascular disease events, including acute myocardial infarction and other forms of coronary heart disease (CHD), stroke, and congestive heart failure; for cardiovascular disease interventions; and for mortality. The first examination took place over two years, from July 2000 - July 2002. It was followed by four examination periods that were 17-20 months in length. Participants have been contacted every 9 to 12 months throughout the study to assess clinical morbidity and mortality.

**The Rotterdam Study (RS)**

The Rotterdam Study (RS) is a prospective, population-based cohort study of determinants of several chronic diseases in older adults. [PMID: 26386597] RS includes 7,983 inhabitants of Ommoord, a district of Rotterdam in the Netherlands, who were 55 years or over. The baseline examination took place between 1990 and 1993, with follow-up visits every 3-5 years. In addition, subjects are actively followed-up for incident disease and mortality. [PMID: 22388767]

**The Study of Health in Pomerania (SHIP)**

The Study of Health In Pomerania [PMID: 20167617] is a prospective longitudinal population-based cohort study in Western Pomerania assessing the prevalence and incidence of common diseases and their risk factors. SHIP encompasses the two independent cohorts SHIP and SHIP-TREND. Participants aged 20 to 79 with German citizenship and principal residency in the study area were recruited from a random sample of residents living in the three local cities, 12 towns as well as 17 randomly selected smaller towns. Individuals were randomly selected stratified by age and sex in proportion to population size of the city, town or small towns, respectively. A total of 4,308 participants were recruited between 1997 and 2001 in the SHIP cohort. Between 2008 and 2012 a total of 4,420 participants were recruited in the SHIP-TREND cohort. Individuals were invited to the SHIP study centre for a computer-assisted personal interviews and extensive physical examinations.

**The Women’s Genome Health Study (WGHS)**

The Women’s Genome Health Study (WGHS) is a prospective cohort of initially healthy, female North American health care professionals at least 45 years old at baseline representing the subset of participants in the Women’s Health Study (WHS) who provided a blood sample at baseline and consent for blood-based analyses, including measurement of plasma biomarkers. The WHS was a 2x2 trial beginning in 1992-1994 of vitamin E and low dose aspirin in primary prevention of cancer and cardiovascular disease with about 10 years of follow-up.  Since the end of the trial, follow-up has continued in observational mode. Additional information related to health and lifestyle were collected by questionnaire throughout the WHS trial and continuing observational follow-up.  Incident cardiovascular disease was ascertained by duplicate physician review of medical records according to WHO criteria.
